# Does the novel coronavirus 2019 like heart more than the other family members of coronaviruses?

**DOI:** 10.34172/jcvtr.2020.27

**Published:** 2020-05-11

**Authors:** Mohammad Mostafa Ansari Ramandi, Mohammadreza Baay, Nasim Naderi

**Affiliations:** ^1^Cardiovascular Diseases Research Center, Birjand University of Medical Sciences, Birjand, Iran; ^2^Rajaie Cardiovascular Medical and Research Center, Iran University of Medical Sciences, Tehran, Iran

**Keywords:** Coronavirus, Heart, Cardiovascular, Severe Acute Respiratory Syndrome (SARS), Middle East Respiratory Syndrome (MERS), COVID-19

## Abstract

The disaster due to the novel coronavirus disease 2019 (COVID-19) around the world has made investigators enthusiastic about working on different aspects of COVID-19. However, although the pandemic of COVID-19 has not yet ended, it seems that COVID-19 compared to the other coronavirus infections (the Middle East Respiratory Syndrome [MERS] and Severe Acute Respiratory Syndrome [SARS]) is more likely to target the heart. Comparing the previous presentations of the coronavirus family and the recent cardiovascular manifestations of COVID-19 can also help in predicting possible future challenges and taking measures to tackle these issues.

## Dear Editor,

The disaster due to the novel coronavirus disease 2019 (COVID-19) around the world has made investigators enthusiastic about working on different aspects of COVID-19.

The patients with coronavirus infection may present with a flu-like symptom rapidly progressing into respiratory distress. This scenario and the respiratory system involvement is common between Middle East respiratory syndrome (MERS), severe acute respiratory syndrome (SARS) and COVID-19. It has also been shown that the presence of cardiovascular comorbidities is accompanied by more severe illness and mortality in all three cases of coronavirus infection.^1-3^

However, although the pandemic of COVID-19 has not yet ended, it seems that COVID-19 compared to the other coronavirus infections (MERS and SARS) is more likely to target the heart.

The term acute cardiac injury has been used to describe the myocardial involvement in COVID-19. It is defined as elevated cardiac biomarkers such as high sensitivity troponin I and accompanied by clinical and imaging findings of cardiac dysfunction. The pathogenesis is still unknown but it seems that the heart may be directly involved during illness and manifested clinically as acute myocarditis.^4^

The prevalence of acute cardiac injury in patients with COVID-19 is thought to be up to 77% in different single and multi-center reports.^5^

There are also case reports presenting patients with a known cardiac problem who manifest with another irrelevant cardiac involvement for example patients with a history of non-ischemic cardiomyopathy or hypertension who have developed cardiogenic shock, in which the criteria of acute myocarditis have been fulfilled.^2^

As far as we investigated and researched, this high prevalence of cardiac injury has not been reported in SARS or MERS, particularly in MERS.

Although in the MERS, the presence of cardiovascular comorbidities was the most important risk factor for death, there are only limited reports regarding the presentation of MERS with myocarditis and/or thrombotic event. The patients with MERS have frequently had medical comorbidities and in most of the case series regarding MERS, respiratory system involvement was the predominant feature.^1^ For example, more than 95% of cases in a study done by Assiri et al had medical comorbidities, from which 28% were cardiac comorbidities.^1^ Among the limited reports, Alhogbani et al in 2016 reported a 60-year-old man with fever, respiratory symptoms and elevated cardiac biomarkers and cardiac MR findings in favor of acute myocarditis.^6^

Acute cardiac injury similar to those reported in COVID-19 has been described in SARS; however, the arterial or venous thrombotic events were more prevalent in these patients.^7^ Umapathi et al reported 5 cases of SARS who presented with cerebrovascular attack complicated with myocardial infarction and disseminated arterial and venous thromboses in one of the cases.^3^

**Figure 1:**
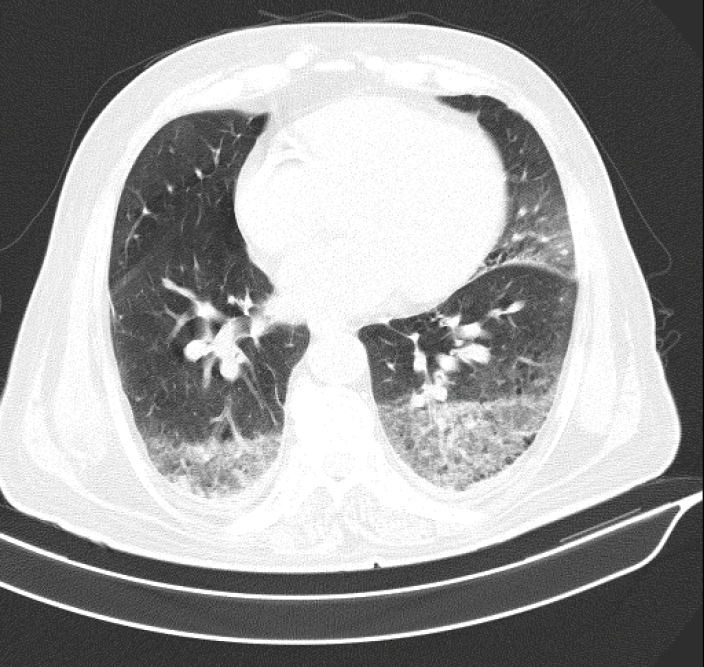
Chest computerized tomography in patient with STEMI and subacute in-stent restenosis.

Finally, we have encountered many patients who present with cardiac-related manifestations and are finally diagnosed as having COVID-19 by polymerase chain reaction (PCR) examination in our center which is a tertiary center for cardiovascular medicine in Tehran, Iran. For instance, we have presented 1 chest computerized tomography (CT) images showing COVID-19 involvement in a PCR positive patient. Figure 1 depicts the chest CT of a patient with ST-segment elevation myocardial infarction (STEMI) and subacute in-stent restenosis.

It seems that the novel coronavirus has a greater tendency to damage the heart in comparison to its older ancestors. The severity of cardiovascular involvement is independent of the respiratory manifestations and the morbidity and mortality will be higher in those with cardiac involvement. The exact mechanisms for cardiovascular involvement in COVID-19 are still unknown. In a report by Tavazzi et al the viral particles were seen in pathologic examination of endomyocardial biopsy samples of a patient with acute cardiac injury and COVID-19. However, these viral particles were more present in the macrophage, perivascular areas and damaged interstitial cells rather than cardiomyocytes.^4^ Currently, besides the controversies regarding the role of Angiotensin-converting enzyme 2 (ACE2) in the pathogenesis of cardiovascular involvement in COVID-19,^5^ the proposed mechanisms for the acute cardiac injury in COVID-19 include the systemic inflammatory response, impaired oxygen utilization and acute ischemic injury.^4,8,9^ Although this virus mainly infects the macrophages and epithelial cells of the respiratory tract, as shown by other investigators,^4,10^ we suggest either migration of macrophages or viremia can infect extrapulmonary organs such as the heart and start an inflammatory cascade in which the cardiac tissue gets damaged.

In conclusion more molecular and pathological studies are needed for the characterization of acute myocardial injury in COVID-19 patients and shed light on the exact reason for this issue. Comparing the previous presentations of the coronavirus family and the recent cardiovascular manifestations of COVID-19 can also help in predicting possible future challenges and taking measures to tackle these issues.

## Competing interests

None.

## Ethical approval

Not applicable.
